# Review of "Animal Models in the Light of Evolution" by Niall Shanks, Ph.D., and C. Ray Greek, M.D

**DOI:** 10.1186/1747-5341-5-12

**Published:** 2010-08-17

**Authors:** Lewis Wolpert

**Affiliations:** 1 Anatomy and Developmental Biology, University College, London, UK

## Abstract

Animal Models in the Light of Evolution provides persuasive evidence that animal models should be used with great caution when applying the results to human diseases. Mice and other model animals are both similar and different, in their biology, to humans.

## 

Animal Models in the Light of Evolution provides persuasive evidence that animal models should be used with great caution when applying the results to human diseases. Mice and other model animals are both similar and different, in their biology, to humans. It is rather technical and not easy reading.

The aim of this book is to question the value of animal experiments for biomedical research, particularly finding chemical treatments for human diseases. Shanks and Greek raise concerns about predictive animal modelling. They particularly question whether animal experiments can prepare the way for trials of medical treatments in humans.

Arguments from evolution are used to show the fundamental differences between, for example, rats and humans, as the organisms are too complex for information about one to be applied to the other. The authors discuss this complexity in terms of dynamical systems theory and its mathematical basis and question the possible similarity of such systems in different mammals. Quite small changes can have significant effects. They use such views to raise doubt on just how much genes contribute to an organism's form and function, but their arguments are themselves complex and hard to follow, and there is no persuasive evidence. It is no mystery that mice can differ significantly from humans in their response to drugs.

Yet it is common for biologists to recognise similarities in the way animals develop, and how they use similar genes and mechanisms. The basic processes in cells are very similar and the problem is to understand just how proteins determine how a cell functions. There is, for example, good reason to believe that the development of limbs in mice, chick, and human are essentially the same. Yet, in support of the author's case, the effect of thalidomide on human embryos that caused loss of proximal structures does not occur in standard experimental animals. There are differences which are not understood. They rightly point out that identical twins do not always suffer from the same diseases - this only occurs about half the time. Discrepancies can be due to differences in development and the influence of the environment.

Although mice are widely used for the study of cancer, their cancers are mainly sarcomas and leukemias, whereas human tumours are mainly carcinomas. It is particularly striking that smoking does not cause cancer in mice. Leading cancer researcher Robert Weinberg has commented: "The preclinical animal models of human cancer, in large part, stink… Hundreds of millions of dollars are being wasted every year by drug companies using these models." In vitro cell and tissue cultures have proven to be powerful investigative tools. Human cancer cell lines are a more reliable and much less costly alternative. Nevertheless studies on animals have provided fundamental information on how cancer develops and spreads.

Moreover animal models have been the way to discover many key features relating to human biology. They were used by Harvey to discover the circulation of the blood, and more recently IVF came from animal studies. Robert Koch (1843-1910) established that specific diseases are caused by specific germs. He saw rod-shaped bacteria in the blood of cows that had died from anthrax and guessed that they caused anthrax. When Koch injected mice with this blood, they also developed anthrax. With Pasteur they also identified the germs causing diphtheria, rabies and the plague. This allowed scientists to develop vaccines for animals and people by using weakened germs.

There are many other examples - human health care has benefited hugely from animal experiments. The authors do not give enough credit to these achievements. Recently real evidence of curing Huntington disease has been found by researchers using mice. Animal studies have also led to the development of drugs to treat epilepsy. Many life-saving surgical procedures, including organ transplantation, heart-valve replacement, coronary artery bypass and open-heart surgery, have been developed using animal models first. But key discoveries in such areas as heart disease, cancer, immunology, anesthesia and psychiatry were in fact achieved through clinical research, observation of patients and human autopsy.

There are however many cases where animal models have been unhelpful. The monkey model of polio misled researchers about polio's mechanism of infection and clinical course, and delayed progress against the disease. Despite their extensive use since the early 1980 s, the predictive validity of animal models for HIV infection in humans remains questionable.

The case that Shank and Greek make about animal models is important and persuasive. Their arguments based on evolution contribute very little.

## Competing interests

The author declares that they have no competing interests.

## Authors' contributions

The author is solely responsible for this manuscript.

## Authors' information

Lewis Wolpert is Emeritus Professor in the Research Department of Cell and Developmental Biology of University College, London. He originally took a degree in civil engineering and carried out research in soil mechanics, and then changed to cell biology at King's College. He has worked on the mechanics of cytokinesis, morphogenesis of the sea urchin embryo, regeneration in hydra, left right asymmetry, and has focussed on pattern formation in limb development. He was made a Fellow of the Royal Society in 1980 and awarded the CBE in 1990. He was made a Fellow of the Royal Society of Literature in 1999. He has also been involved in interacting with the public in relation to science.

